# T-wave loop area from a pre-implant 12-lead ECG is associated with appropriate ICD shocks

**DOI:** 10.1371/journal.pone.0173868

**Published:** 2017-03-14

**Authors:** Joachim Seegers, Katerina Hnatkova, Tim Friede, Marek Malik, Markus Zabel

**Affiliations:** 1 Department of Cardiology and Pneumology, Division of Clinical Electrophysiology, University Medical Center Göttingen, Göttingen, Germany; 2 Department of Internal Medicine II, Division of Cardiology, Section of Electrophysiology, University Hospital Regensburg, Regensburg, Germany; 3 National Heart and Lung Institute, Imperial College, London, United Kingdom; 4 Department of Medical Statistics, University Medical Center Göttingen, Göttingen, Germany; 5 DZHK (German Centre for Cardiovascular Research), partner site Göttingen, Göttingen, Germany; University of Oxford, UNITED KINGDOM

## Abstract

**Aims:**

In implantable cardioverter-defibrillator (ICD) patients, predictors of ICD shocks and mortality are needed to improve patient selection. Electrocardiographic (ECG) markers are simple to obtain and have been demonstrated to predict mortality. We aimed to assess the association of T-wave loop area and circularity with ICD shocks.

**Methods:**

The study investigated patients with ICDs implanted between 1998 and 2010 for whom digital 12-lead ECGs (Schiller CS200 ECG-Network) of sufficient quality were obtained within 1 month prior to the implantation. T-wave loop area and circularity were calculated. Follow-up data of appropriate shocks were obtained during ICD clinic visits that included reviews of device stored electrograms.

**Results:**

A total of 605 patients (82% males) were included; 68% had ischemic cardiomyopathy and 72% were treated for primary prevention. Over 3.8±1.4 years of follow-up, 114 patients (19%) experienced appropriate shock(s). Those with smaller T-wave loop area received fewer shocks (TLA, hazard ratio, HR, per increase of 1 technical unit, 0.71; [95% confidence interval, 0.53–0.94]; *P* = 0.02) and those with larger T-wave loop circularity (TLC) representing rounder T wave loop received more shocks (HR per 1% TLC increase 2.96; [0.85–10.36]; *P* = 0.09). When the quartile containing the largest TLA and TLC values, respectively, were compared to the remaining cases, TLA remained significantly associated with fewer and TLC with more frequent shocks also after multivariate adjustment for clinical variables (HR, 0.59 [0.35–0.99], *P* = 0.044; and 1.64 [1.08–2.49], *P* = 0.021, respectively).

**Conclusions:**

The size and shape of the T-wave loop calculated from pre-implantation 12-lead ECGs are associated with appropriate ICD shocks.

## Introduction

Implantable cardioverter defibrillators (ICD) have been shown to improve survival.[1,2] Current guidelines recommend their use for primary and secondary prevention of sudden cardiac death (SCD).[2] However, a large number of patients never receive appropriate device therapy.[3] Noninvasive risk stratification beyond left ventricular ejection fraction remains challenging[4] and has not been reflected in clinical routine. Electrocardiographic (ECG) markers are simple to obtain and have been shown to predict mortality in clinically well defined populations including elderly subjects, myocardial infarction survivors and cardiovascular disease patients, as well as characterize patients with previously documented ventricular tachycardia and fibrillation.[5–8] These studies reported, among others, prognostic and diagnostic value of the three-dimensional QRS-T angle and of the characteristics of the T loop area (TLA), related to the level of repolarization synchrony over both ventricles. The latter represents a disturbance of repolarization and thus electrical vulnerability and may therefore be a promising tool for risk stratification of SCD. Studies focusing on measurements of the QRS complex duration showed contradicting results.[9]

We therefore hypothesized that T wave loop descriptors will be associated with appropriate shocks in a large single-center ICD population with long-term follow-up. In addition, we investigated the association of QRS duration with appropriate ICD shocks. QRS duration and the so-called total cosine of the of three-dimensional R-to-T angle (TCRT), an expression of the QRS-T angle,[5] are well known predictors of mortality in cardiac patients. We therefore also aimed at confirming their power to predict mortality in the ICD population.

## Methods

### Patients

A registry was compiled of consecutive patients undergoing cardioverter-defibrillator (ICD) or cardiac resynchronization defibrillator implantation at University Medical Center Göttingen, Germany, for guideline recommended indications[2] between 1998 and 2010. For the purpose of this study, patients were selected if a technically valid digital 12-lead surface ECG (Schiller Inc., Baar, Switzerland) was available on the hospital-wide network (Schiller SEMA/SDSDB database) obtained during the last month prior to the first ICD implantation. Of 1272 registry patients, 605 (48%) fulfilled this criterion. If patients had more than one ECG available within this month, the ECG recorded closest to the implantation date was selected for the analysis. The ECGs used for analysis were obtained 9.5±8.1 days prior to the ICD implantation procedure. Another 53 patients had a digital ECG available, but the automatic detection of the end of the T wave failed due to flat or biphasic T waves. The remaining patients had their preoperative ECG on the ward, but not stored digitally. Data were analyzed retrospectively using de-identified data.

### T-wave morphology descriptors

Digitally exported 10-second 12-lead surface ECGs were sent to Imperial College London for blind analysis. TLA and TCRT were determined automatically using previously described algorithms and analysis techniques.[5,6,10] In brief, TLA measures the area of the T wave loop projected into the dominant plane of the loop. The T wave circularity (TLC), previously also called “normalized TLA”, describes the relationship between the area of the loop and the length of the line of the loop. TCRT determines the integral-measured cosine of the three-dimensional angle between the depolarization and the repolarization vectors. The calculations were made in representative QRS-T complexes obtained by sample-by-sample voltage medians of aligned superimposed beats of the 10 second recordings. [11]

Algorithmic QRS duration measurement was also performed in superimposed representative complexes of all 12 leads and visually checked with manual correction where necessary. The measurement was made blindly in respect of the follow-up outcome of the patients.

### Device programming and interrogation

Devices of three different ICD manufacturers were implanted (Biotronik, Berlin/Germany; Boston Scientific, Natick, MA/USA, formerly CPI, Guidant, St Paul, MN/USA; and Medtronic Inc., Minneapolis, MN/USA). The parameters of tachycardia detection varied over time according to available knowledge and recommendations available at the implantation. ICD interrogation and programming was done by a single ICD technician over the whole study period. Detection rates and detection times were programmed more conservatively in the later years. In general, ventricular fibrillation was detected with heart rates above 210-230 bpm lasting for 1 to 2.5 s or 12/16 to 18/24 beats. Ventricular tachycardia was identified with heart rates above 170 bpm, lasting 2.5 to 5 s or 16 to 24 beats. ICD therapy incorporated antitachycardia pacing during charging in the ventricular fibrillation zone, if applicable, followed by shocks of maximal energy. Ventricular tachycardias were treated by 6–12 antitachycardia pacing cycles of burst and ramp trains followed by shocks of maximal energy. In patients with an ICD implanted for secondary prevention of ventricular tachyarrhythmia, individual programming reflected previously documented arrhythmias. Algorithms for improved differentiation of supraventricular arrhythmias (onset, stability, Biotronik “S.M.A.R.T.”, Medtronic “Wavelet” or “PRLogic”, Boston Scientific “RhythmID”) were activated if clinically appropriate.

### Follow up and endpoints

All-cause mortality and first appropriate ICD shock (shock for ventricular tachycardia or fibrillation as judged by the ICD’s episode documentation of intracardiac signals) were prospectively defined as registry endpoints. The majority of patients (approximately 70%) were followed at the outpatient ICD clinic of the University Medical Center Göttingen. If subjects were also followed outside this ICD clinic, the patient’s treating cardiologist and/or the patient were contacted by letter questionnaire and/or telephone. Available EGMs were reviewed clinically, the review was repeated for research purposes by one of the authors (M.Z.). All-cause mortality was assessed based on hospital data, information from other hospitals, the patients’ general practitioner, or governmental registry of deaths.

### Statistical analysis

Variables are presented as mean±SD for continuous variables and as proportions for categorical variables. T-wave morphology descriptors and QRS duration were not normally distributed upon Kolmogorov-Smirnov test and therefore reported as median and interquartile range (IQR) and compared using Mann-Whitney U-Test. For further analysis, T-wave morphology descriptors and QRS duration were dichotomized by appropriate quantiles (e.g. median, first or third quartiles) and compared using cumulative incidence functions including death as competing risk. Fine and Gray’s proportional subdistribution hazards regression models were fitted to determine the effects of multiple variables on the time-to-first appropriate shock which is censored by death as terminal competing event.[12] Clinical variables significantly associated with ICD shocks (univariable P<0.05) were included in the multivariable model. The assumption of proportional subdistribution hazards was checked visually by plotting scaled Schoenfeld residuals against follow-up time.[13] Cox regression models were used to investigate association with mortality. Relationship of the TLA, TLC and QRS duration were expressed in terms of the Pearson correlation coefficient. Variables with statistically significant effects (*P*<0.05) were included in the final model. A p-value <0.05 was considered statistically significant.

## Results

### Patients

The mean age at ICD implantation was 65±11 years, 498 of 605 (82%) patients were males (Table 1). Ischemic and non-ischemic cardiomyopathy was diagnosed in 414 (68%) and 173 (29%) patients, respectively, the remaining 18 (3%) patients had ion channel disease or idiopathic arrhythmias. The majority of the cohort had a typical heart failure medication; 528 (90%) patients were treated with angiotensin converting enzyme inhibitors or angiotensin receptor blockers, 545 (93%) received β-blockers, diuretics were used in 432 (74%) patients, mineralocorticoid receptor antagonists in 298 (51%) patients, and digitalis glycosides in 183 (31%) patients. A total of 96 of 605 patients (16%) were treated with amiodarone at implantation. At implantation, 234 (39%) patients had a history of atrial fibrillation, renal dysfunction (as evidenced by an elevated serum creatinine) was diagnosed in 253 (42%) patients; 165 (27%) patients suffered from diabetes, 98 (16%) from chronic obstructive pulmonary disease, and 51 (9%) from peripheral arterial disease.

**Table 1:** Baseline characteristics of the 605 patients.

Age (years)	65±11
Sex category (male)	498 (82%)
Body mass index (kg/m^2^)	27±5
NYHA functional class >II	302 (51%)
Primary prophylactic indication	437 (72%)
biventricular ICD	239 (40%)
Ischemic cardiomyopathy	414 (68%)
LV ejection fraction (%)	28±10
QRS duration (ms)	123±21
estimated glomerular filtration rate (ml/min/1.73m^2^)	65±22
β-Blocker	545 (93%)
Amiodarone	96 (16%)
History of atrial fibrillation	234 (39%)

ICD implantable cardioverter-defibrillator, NYHA New York Heart Association

### Outcomes

All patients were followed up for to 5 years (average 3.8±1.4 years). A total of 137 patients (23%, 6.1% annually) died, and 114 (19%, 5.0% annually) received an appropriate ICD shock for ventricular arrhythmias. The mean cycle length of arrhythmias associated with the first appropriate shock was 273±60 ms. At least one inappropriate shock was delivered in 53 (8.8%) patients, 21 of whom were also treated with appropriate shock(s).

### T wave descriptors and ICD shocks

**Fig 1:**
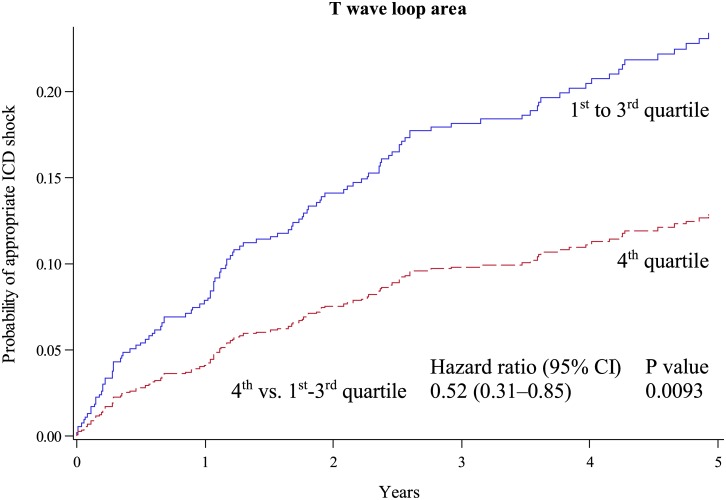
Rate of first appropriate ICD shock by T wave loop area. Cumulative incidence functions for the probability of appropriate ICD shock according to 25% of patients with the largest loops (red dotted line) vs. 75% of patients with the smaller loops (blue line).

**Fig 2:**
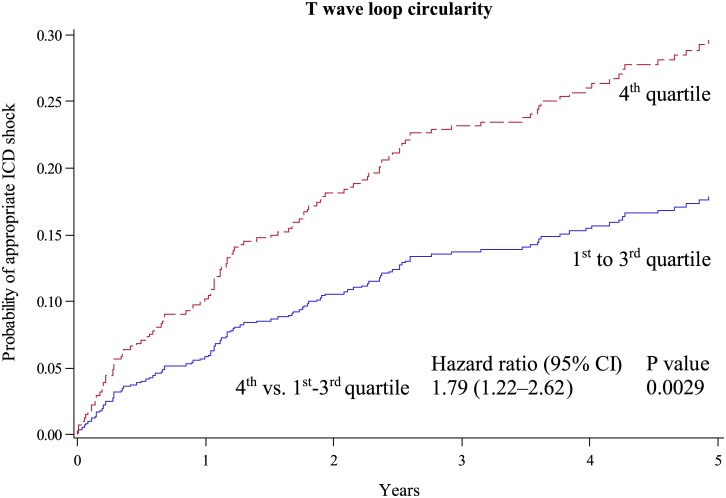
Rate of first appropriate ICD shock by T wave loop circularity. Cumulative incidence functions for the probability of appropriate ICD shock according to 25% of patients with the least compact loops (highest T wave loop circularity, red dotted line) vs. 75% of patients with more compact loops (blue line).

The results of the T wave descriptors and QRS duration are presented in Table 2: Patients with a larger size of the loop were at lower risk for appropriate ICD shock during follow-up; patients with a rounder and more open loop (as indicated by a higher TLC) had a higher incidence of shocks by trend. TLA and TLC values, respectively were grouped in four equally sized groups using quartiles (for TLA: 1^st^ quartile, < 0.0347 t.u.; 2^nd^ quartile, 0.0347 to 0.0847 t.u.; 3^rd^ quartile, 0.0848 to 0.7225 t.u.; and 4^th^ quartile > 0.7226 t.u.; for TLC 1^st^ quartile, < 0.343; 2^nd^ quartile, 0.343 to 0.452; 3^rd^ quartile, 0.453 to 0.588; and 4^th^ quartile > 0.588). The estimated mean 5-year ICD shock incidence was the lowest in the highest quartile of TLA values and the highest in the highest quartile of TLC values. In addition, the population was also divided into two groups selecting the upper quartile vs. the remaining patients (the 4^th^ quartile of TLA or the 4^th^ quartile of the TLC in one group and the remaining three quartiles in the other group). In the 25% of patients having the largest loops (vs. the 75% of patients with smaller loops), a lower incidence of shocks was found (Fig 1). In the 25% of patients with the least compact loops (i. e. the highest TLC values), the incidence of shocks was higher (Fig 2).

**Table 2:** T wave morphology descriptors and QRS duration.

	TLA	P value	TLC	P value	TCRT	P value	QRS	P value
*Median (IQR) values*								
with shock	0.07 (0.03; 0.32)	0.046	0.48 (0.32; 0.65)	0.11	-0.81 (-0.94; -0.02)	0.74	117 (107; 132)	0.23
without shock	0.10 (0.04; 0.84)		0.44 (0.34; 0.58)		-0.72 (-0.92; -0.08)		121 (107; 137)	
*Shock association*								
HR (95% CI) per 1 unit	0.71 (0.53–0.94)	0.02	2.96 (0.85–10.36)	0.09	1.09 (0.75–1.57)	0.65	0.99 (0.98–1.00)	0.12
*5-year ICD shock incidence*								
1st quartile	23%	0.06	22%	0.007	27%	0.13	21%	0.42
2nd quartile	25%		13%		18%		25%	
3rd quartile	22%		19%		17%		20%	
4th quartile	13%		30%		21%		16%	
*5-year ICD shock incidence*								
1st to 3rd quartile	23%	0.009	18%	0.003	21%	0.96	22%	0.16
4th quartile	13%		30%		21%		16%	

IQR interquartile range

Association of clinical variables and ICD shocks was investigated using Fine and Gray regression model (Table 3). Both variables, TLA and TLC, remained significant when entered into a multivariate Fine and Gray regression model together with all significant variables from Table 3, i.e. mode of prevention, LV ejection fraction, use of amiodarone and history of atrial fibrillation (Table 4). Although statistically significant, the correlation between TLA and TLC was weak (Pearson correlation coefficient of 0.35). Neither TLA nor TLC were associated with mortality (*P* = 0.45 and *P* = 0.78 for continuous variable and *P* = 0.82 and *P* = 0.56 for highest vs. remaining quartiles, respectively).

**Table 3:** Fine and Gray regression for appropriate shock.

	unadjusted Hazard Ratio [95% CI]	*P*
Age (per 10 years)	0.96 [0.82–1.12]	0.576
Sex category (male)	1.47 [0.83–2.59]	0.187
Body mass index (per 10 kg/m^2^)	1.17 [0.78–1.76]	0.439
NYHA functional class >II	0.85 [0.59–1.23]	0.394
Primary prophylactic indication	0.66 [0.45–0.97]	**0.035**
biventricular ICD	0.87 [0.60–1.27]	0.463
Ischemic cardiomyopathy	1.21 [0.80–1.81]	0.365
LV ejection fraction (per 10%)	1.23 [1.04–1.47]	**0.015**
QRS duration (per 10 ms)	0.93 [0.85–1.02]	0.121
estimated glomerular filtration rate (per 10 ml/min/1.73m^2^)	0.99 [0.90–1.08]	0.841
β-Blocker	1.29 [0.57–2.93]	0.542
Amiodarone	1.78 [1.17–2.72]	**0.007**
History of atrial fibrillation	1.58 [1.10–2.28]	**0.014**

ICD implantable cardioverter-defibrillator, NYHA New York Heart Association

**Table 4:** Fine and Gray regression for appropriate shock adjusted for clinical variables (see text; The upper and the remaining quartiles of TLA and TLC, respectively, were compared; TLA and TCL were not entered into the same model).

	Adjusted Hazard Ratio [95% CI]	*P*
T-wave loop area 4^th^ quartile	0.59 [0.35–0.99]	0.044
T wave circularity 4^th^ quartile	1.64 [1.08–2.49]	0.021

CI confidence interval

**Fig 3:**
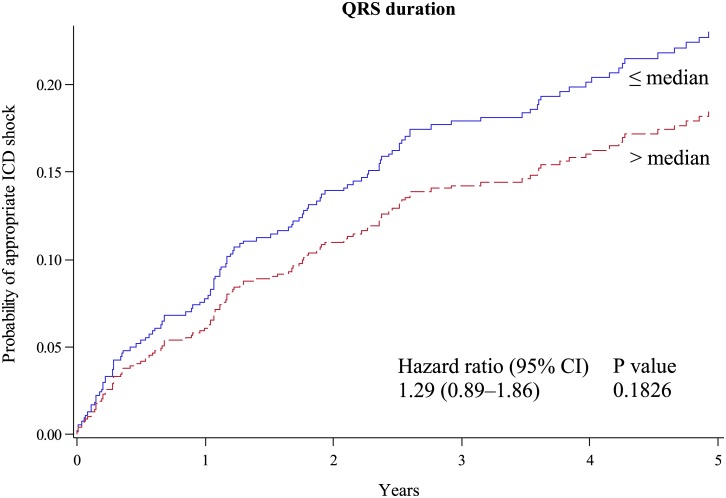
Rate of first appropriate ICD shock by QRS duration. Cumulative incidence functions for the probability of appropriate ICD shock according to QRS duration above (red dotted line) and below (blue line) the median.

Although QRS duration was not significantly associated with ICD shocks (*P* = 0.12, Table 3), we also investigated possible interactions between QRS duration and the T-wave parameters: Half of our patients had a QRS duration of more than 120 ms, and 126 patients had typical left bundle branch block. The correlation between QRS duration and TLA was moderate (Pearson correlation coefficient of 0.68), correlation between QRS duration and TLC was weak (-0.24). The estimated mean 5-year ICD shock incidence in the quartiles of QRS duration was comparable in all quartiles (Table 2). When the population was divided by the QRS duration median (120 ms), no significant difference of ICD shock incidence was observed (23% vs. 18%, unadjusted *P* = 0.18, Fig 3) and the same was observed, when the population was divided into the quartile with the widest QRS duration compared to the remaining three quartiles with smaller QRS width together (Table 2). In contrast, QRS duration was associated with mortality (HR per 10 ms increase of duration, 1.10; 95%-CI, 1.02–1.18; *P* = 0.018).

To ensure the validity of the ECG dataset, TCRT was analyzed and, as expected, found to be lower in patients who died during follow up (HR for mortality per unit decrease, 1.98; 95%-CI, 1.37–2.87; *P* = 0.00029); however, no association with appropriate ICD shocks was observed, neither, if analysed as a continuous variable, nor using quartiles (Table 2).

## Discussion

Our data show that T-wave loop area and T wave circularity but not QRS duration are associated with appropriate shocks in ICD patients. Patients with a larger T wave loop have a lower incidence of shocks, while patients in whom the T wave loop is more round and more open (higher TLC) have significantly more shocks. Known correlations between TCRT or QRS duration and mortality were confirmed in this dataset, supporting the validity of the analyzed ECG recordings.

### Patient population and events

Altogether, 19% of our patients (or 5.0% annually) received appropriate ICD shocks. This is similar to the rate observed in the Sudden Cardiac Death in Heart Failure Trial (SCD-HeFT; 5.1%)[1] but less than in the Multicenter Automatic Defibrillator Implantation Trial-II (MADIT-II; 10.7%).[14] Similarly, the observed all-cause mortality of 6.0% per year in our study was comparable to SCD-HeFT (5.8%)[1] but lower compared to MADIT-II (8.5%).[14] ICD shocks for malignant ventricular arrhythmia are considered here as a surrogate outcome for arrhythmic death. Our ICD programming aimed to avoid unnecessary ICD shocks using a moderate detection delay and a large number of antitachycardia pacing trains in the ventricular tachycardia zone. This was not considered an endpoint, and eventually resulted in a low annual shock rate. A previous study found twice as many ICD shocks occurring in the ICD group compared to arrhythmogenic death in the non-ICD control group,[15] thus, the ICD shock rate reported in previous studies may overestimate the number of fatal arrhythmias, as a large number of shocks is delivered for non-fatal arrhythmia.[16] Therefore, we consider it justified not to count antitachycardia pacing as an endpoint. Although the use of appropriate ICD shocks as a surrogate of arrhythmic mortality in ICD patients has been criticized,[15] there is no alternative choice in evaluating the incidence of malignant arrhythmias in ICD patients. Appropriate ICD shocks may overestimate but definitely do not underestimate a risk of arrhythmogenic mortality had the patient not been implanted an ICD.

### T-wave loop area and TCRT

TLA and TLC are both descriptors of the repolarization over the entire ventricles. It seems reasonable to hypothesize that the size of the loop is, in principle, a more accurate and lead independent expression of flat T waves; generally, flat T waves produce a smaller loop. Likewise, a larger TLA represents a more synchronized repolarization over the complete ventricles. The shape of the loop, measured by TLC, describes the repolarization dispersion of the repolarization across the ventricles, and a large and open loop more like a circle resulting in a high TLC corresponds to a more diverse repolarization across the ventricles as recently shown [17]. It was previously reported that a round T wave loop is present in ischemic heart disease.[8] In addition, the relationship of repolarization abnormalities and arrhythmia has previously been shown.[18] It is therefore reasonable that patients with well above average size of the T wave loop have significantly lower incidence of shocks (13% vs. 23%) and patients with a round shape of the T wave loop a higher incidence of shocks (30% vs. 18%), if the quartile with the highest values was compared to the remaining population each. Hence, although there is no direct comparison of our finding with existing literature, the observations are in principal agreement with previous reports. Of interest, TLA and TLC are not associated with total mortality in our ICD patient cohort suggesting that variables associated with ICD shocks are not necessarily associated with mortality.

T-wave morphology parameters have been investigated earlier in patients without ICD therapy: In the Finnish population-based Health 2000 Study, T-wave morphology dispersion and TRCT were identified as independent predictors of SCD risk in 5618 adults of a general population sample followed over 7 years (annual SCD event rate 0.16%).[19]

In a cohort of 280 post-myocardial infarction patients, TCRT was an independent predictors of mortality.[6] In contrast to our results, no difference was reported for TLC in patients with and without arrhythmic events during follow-up. This difference might have been contributed by the use of re-scanned paper-printed ECG tracings in the previous study.[6] Also, in the previous study, patients were younger (average 59 years vs. 65 years), left ventricular ejection fraction was higher (47% vs. 28%) and fewer patients were treated with beta- blockers (82% vs. 93%). Moreover, since the previous study used older data, the rate of reperfusion therapy in myocardial infarction was low (55%) whereas acute percutaneous coronary intervention was standard of care in our patients since 2003. In contrast, QRS duration, a well known marker of all-cause mortality[20] as observed in our analysis, was not associated with appropriate ICD shocks in our cohort confirming results reported from a subanalysis of SCD-HeFT.[9] As we were able to show an association of mortality and QRS duration as well as TCRT in our dataset, the validity of the analyzed ECG can be strongly assumed. Of note, these mortality markers were not associated with appropriate ICD shocks as TLA und TLC were and vice versa.

### Impact of improved patient selection for ICD therapy

Four out of every five deceased patients in our cohort did not receive any appropriate ICD shock and therefore had no benefit from the ICD therapy. In addition, the risk of inappropriate shock still remains a dilemma. ICD therapy in general raises questions regarding patient’s comfort, outcome and the health care system: From the patients’ perspective, the potential complications of the implant procedure and of the device and especially lead performance have potentially substantial impact on the quality of life. In addition, it has been recently highlighted by two randomized trials investigating ICD programming that the presence of a device is potentially harmful, as pro-arrhythmic effects of ICD therapy including inappropriate ICD shocks may provoke nonfatal ventricular arrhythmia.[3] Thus, avoiding ICD implant in a patient who will not need it is likely to prevent short- and long-term serious adverse effects. An improved patient selection will enhance the cost-effectiveness of the ICD therapy.[21] Moreover, a significant number of patients die suddenly without satisfying ICD indication by current guidelines.[22] It is therefore worthwhile and necessary to identify predictors of life-saving ICD shocks in order to improve patient selection. A prospective investigation is warranted to investigate the hypothesis that both variables described in this study demonstrate usefulness in improving patient selection for ICD therapy e.g. as a continuous variable entered in a multivariate risk score for prediction of appropriate shock in ICD patients.

### Limitations

Retrospective data analysis, although made blindly, can only be hypothesis generating. Only approximately 50% of our total cohort had an analyzable digital ECG for the purpose of this study, which may introduce the potential for additional bias within the results. Nevertheless, the majority of patients without analyzable ECGs have not had the digital signals captured. It is therefore unlikely that our results were skewed because of peculiar abnormalities in patients whose ECG data were not analyzed. Factors like LV remodeling over time resulting in changes in propensity for arrhythmia are not reflected in our analysis focusing on an ECG value obtained at a single point of time. In addition, ischemic, non-ischemic cardiomyopathy and ion channel diseases or idiopathic arrhythmias may have a different arrhythmogenic substrate.

Furthermore, ICD programming influences the number of ICD shocks delivered for ventricular arrhythmias and could not have been uniform in our cohort enrolled over 7 years. Importantly, the ICD clinic technician never changed over this period, ensuring consistent ICD programming at discharge with the best known programming at the time. In addition, anti-tachycardia pacing, which was not considered as an endpoint, might have influenced the occurrence of ICD shocks in some cases. However, in patients having data regarding anti-tachycardia pacing available, it was delivered in 12% of patients without appropriate ICD shock and 49% of patients with appropriate ICD shock. Nevertheless, appropriate AICD shock is not systematically a sudden cardiac death surrogate. Sustained and non-sustained ventricular tachycardia, ventricular fibrillation are three distinct arrhythmias that may be associated with different outcomes.

## Conclusion

The T-wave loop area and circularity calculated from the 12-lead surface ECG are independently associated with appropriate ICD shocks.

## Abbreviations

CI: confidence interval
ECG: electrocardiogram
HR: hazard ratio
ICD: implantable cardioverter-defibrillator
IQR: interquartile range
MADIT: Multicenter Automatic Defibrillator Implantation Trial
NYHA: New York Heart Association
SCD: sudden cardiac death
SCD-HeFT: Sudden Cardiac Death in Heart Failure Trial
TCRT: total cosine of the of three-dimensional R-to-T angle
TLA: T-wave loop area
TLC: T-wave loop circularity
